# Rapid and Cost-Efficient Enterovirus Genotyping from Clinical Samples Using Flongle Flow Cells

**DOI:** 10.3390/genes10090659

**Published:** 2019-08-29

**Authors:** Carole Grädel, Miguel Angel Terrazos Miani, Maria Teresa Barbani, Stephen L Leib, Franziska Suter-Riniker, Alban Ramette

**Affiliations:** 1 Institute for Infectious Diseases, University of Bern, CH-3012 Bern, Switzerland; 2 Graduate School for Cellular and Biomedical Sciences, University of Bern, CH-3012 Bern, Switzerland

**Keywords:** enterovirus, NGS, nanopore sequencing, diagnostics

## Abstract

Enteroviruses affect millions of people worldwide and are of significant clinical importance. The standard method for enterovirus identification and genotyping still relies on Sanger sequencing of short diagnostic amplicons. In this study, we assessed the feasibility of nanopore sequencing using the new flow cell “Flongle” for fast, cost-effective, and accurate genotyping of human enteroviruses from clinical samples. PCR amplification of partial *VP1* gene was performed from multiple patient samples, which were multiplexed together after barcoding PCR and sequenced multiple times on Flongle flow cells. The nanopore consensus sequences obtained from mapping reads to a reference database were compared to their Sanger sequence counterparts. Using clinical specimens sampled over different years, we were able to correctly identify enterovirus species and genotypes for all tested samples, even when doubling the number of barcoded samples on one flow cell. Average sequence identity across sequencing runs was >99.7%. Phylogenetic analysis showed that the consensus sequences achieved with Flongle delivered accurate genotyping. We conclude that the new Flongle-based assay with its fast turnover time, low cost investment, and low cost per sample represents an accurate, reproducible, and cost-effective platform for enterovirus identification and genotyping.

## 1. Introduction

Enteroviruses are RNA viruses and among the most common viruses infecting humans. Enterovirus infections are associated with a broad range of clinical symptoms including mild respiratory illness to severe neurological diseases [1]. Their genomes have high variability due to frequent mutations and recombinations and more than 100 genotypes are known to infect humans [2,3]. The surveillance of circulating genotypes and detection of new emerging genotypes must be conducted at regular intervals. Molecular diagnostic tests for genotyping of enteroviruses usually target the *VP1* region, which is known to correlate with viral serotype [4], by Sanger sequencing the PCR amplified gene. Sanger sequencing is still the standard for enterovirus amplicon sequencing as next generation sequencing (NGS) approaches tend to have longer turnaround times and generally require larger capital investments [5].

The MinION sequencer from Oxford Nanopore Technologies (ONT) is a portable device that allows real-time sequencing and low capital cost investment. Thousands of single nanopores are arrayed on individual synthetic polymer membranes in a single flow cell, and nucleic acid molecules loaded onto the flow cell migrate to and through nanopores by following an electric potential [6]. While single nucleic molecules pass through individual nanopores at a rate determined by an engineered motor protein, the current at each nanopore is modified as a function of the actual molecule composition [7], and those current fluctuations are recorded and converted into base sequences by using a recurrent neural network algorithm [8]. Noticeably, for amplicon sequencing applications, sequencing cost can be further reduced by multiplexing several samples together, i.e., by adding short indexed adaptors (i.e., barcodes) specifically to sequences of each sample, so that the sample origin of each read can be identified. MinION sequencing has been used for sequencing viral amplicons before [9,10,11]. A standard MinION flow cells can reach a total output of 10–20 Gb of data within 48 h and enough sequencing depth can be achieved within a few minutes of sequencing to produce a consensus sequence whose accuracy is then >99% nt identical to its Sanger sequenced counterpart [10]. Recently, ONT has released a new type of flow cell called “Flongle” (R9.4.1 nanopores), which is smaller and cheaper than the current flow cell (SpotON flow cell Mk I, R9.4.1 nanopores). As a small consumable flow cell, a Flongle is mounted onto a Flongle adapter that contains the ONT proprietary sensor array and is compatible with MinION and GridION sequencing devices. A Flongle harbors 126 sequencing channels instead of the standard 512 channels and is typically designed for applications which require less sequencing output than standard flow cells.

The goal of this study was to evaluate whether amplicon sequencing using Flongles was suitable for enterovirus genotyping from diagnostic samples. We tested whether several tens of clinical samples could be multiplexed and sequenced on single Flongle flow cells. We compared nanopore sequencing accuracy to that of Sanger sequencing using repeated assays, and established turnover time and cost of the assays in order to provide first insights on potential clinical applications of Flongle-based sequencing.

## 2. Materials and Methods 

### 2.1. Sample Description and Preparation

Clinical samples originating from the Insel hospital in Bern, Switzerland, were referred to the Institute for Infectious Diseases (IFIK), Bern, for enterovirus diagnostics. Ethics approval was granted by the Swiss Ethics committee on research involving humans on 8 March 2018 to conduct sequencing of enteroviruses in clinical samples stored in the IFIK biobank (BASEC-Nr: Req-2018-00158). In the standard routine diagnostic procedure, all samples are tested by enterovirus-specific real-time PCR (RT-PCR). Cell culture on six cell lines (namely BGMK, Caco-2, Vero, MRC-5, A-549, and RD) is additionally performed for virus detection in stool and swab samples. Supplementary information about the samples can be found in Table S1.

**Figure 1:**
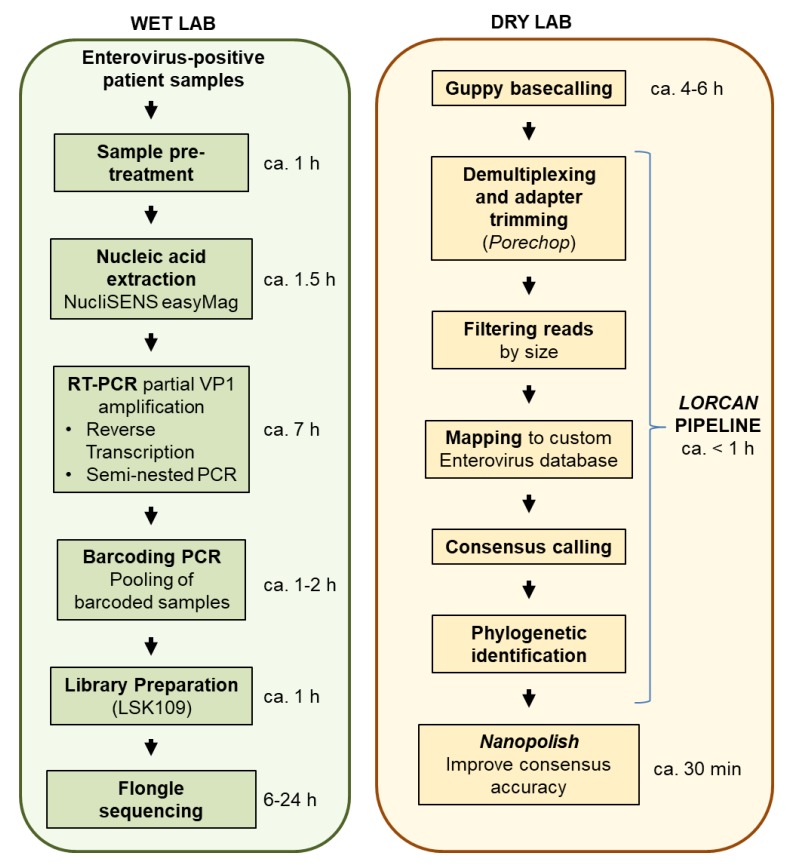
Flow chart of the main steps involved in enterovirus genotyping using Flongle flow cells. For each step, approximate time estimates are provided. The dry lab time estimations are based on using the *LORCAN* pipeline on a Linux server (2.4 GHz Intel Xeon E5 processors) and running *Guppy* 3.1.5 (high accuracy base-calling mode), *LORCAN*, and *nanopolish* with 16, 20, and 20 threads, respectively.

For stool samples, samples were mixed using a sterile pipette and small stool amounts (about 0.6 cm diameter or 1 mL of liquid stool) were resuspended in 4 mL of viral transport medium [12] (Figure 1). Five to ten glass beads (2 mm diameter; Merck AG, Zug, Switzerland) were added and the suspension vortexed for 30 s. After centrifugation for 5 min at 3350 g, the supernatant was sterile filtered with 20% penicillin/streptomycin (Biochrom, Berlin, Germany). Respiratory swabs were collected in viral transport medium and extracted without additional processing.

### 2.2. RT-PCR

Clinical samples were all tested positive for enterovirus presence using a one-step assay with the AgPath-ID one-step kit (Thermo Fisher Scientific, Reinach, Switzerland). The RT-PCR was performed with primer and probes published previously [13], which were synthesized at Microsynth AG (Balgach, Switzerland). The 80 μL RT-PCR/PCR mix contained 40 μL of 2× RT-PCR buffer, 3.2 μL of 25× RT-PCR enzyme mix, 1.8 μL primer/probe-mix, and 5 μL enhancer, to which a total of 30 μL of the RNA extract was added. On a COBAS TaqMan48, the reverse transcription was set at 45 °C (2 min), initial denaturation at 95 °C (2 min), and the amplification was performed for 45 cycles with the following conditions: 95 °C for 15 s, 60 °C for 20 s, and 60 °C for 25 s.

### 2.3. Nucleic Acid Extraction, PCR Amplification, Sanger Sequencing-Based Genotyping

Nucleic acids were extracted from the previously treated samples with NucliSens EasyMAG (bioMérieux, Geneva, Switzerland) as per the manufacturer’s instructions. Extractions were made from 200 μL (or 400 μL) sample volume and eluted in 25 μL (or 50 μL), or from 200 μL sample volume containing 2.5 μL of carrier RNA (Qiagen AG, Hombrechtikon, Switzerland), and finally eluted in 110 μL elution buffer. Reverse transcription and PCR amplification of the partial *VP1* gene were performed according to the semi-nested PCR protocol described by Nix et al. [14], with minor modifications that consisted of using the Superscript IV enzyme (ThermoFisher) and ZymoTaq polymerase (Lucerna Chem, Luzern, Switzerland) for the PCR reactions. The universal tail sequences provided by Oxford Nanopore Technologies (forward primer: 5’-TTTCTGTTGGTGCTGATATTGC-3’, reverse primer: 5’-ACTTGCCTGTCGCTCTATCTTC-3’) were added to primers AN89 and AN88 during primer synthesis at Microsynth AG (Balgach, Switzerland). The size of the PCR product was about 420 bp (including primer tails; exact length depended on genotype). PCR products were purified using Agencourt AMPure XP magnetic beads (Beckman Coulter, Nyon, Switzerland) according to the manufacturer’s instructions for PCR clean up. DNA quantification was done using a dsDNA broad range assay kit on a Qubit fluorometer 3.0 (Thermofisher, Reinach, Switzerland). Purified PCR products were sent for Sanger sequencing at Microsynth (Balgach, Switzerland). Forward and reverse strands were assembled into consensus sequences using SeqMan Pro (DNAStar, Madison, WI, USA). Genotypes were determined by BLASTN similarity analysis against all enterovirus sequences present in GenBank. Genotype confirmation was obtained by submitting consensus sequences to the enterovirus genotyping tool *RIVM* [15,16]. 

### 2.4. Nanopore Library Preparation and Sequencing

In order to produce homogeneous starting material, PCR amplicons were produced only once per sample. Each PCR plate always included negative (non-template water) controls, which did not produce visible bands on agarose gels. PCR negative tubes were not included further in the sequencing run because they did not meet the minimum DNA concentration needed for nanopore library preparation. Amplicon samples were barcoded using the PCR barcoding expansion 1-96 (EXP-PBC096) kit from ONT according to the manufacturer’s instructions (Supplementary Text 1). Equal quantities of each barcoded amplicon were pooled together. A total of 300 ng of the pooled barcoded amplicons was prepared using a ligation sequencing kit (SQK-LSK109) according to the manufacturer’s instructions for Flongle application (Supplementary Text 1) and the total amount of nucleic acids loaded onto the Flongle flow cells (FLO-FLG106) was of 95 ng (run 1) and 30.4 ng (run 2). The MinKNOW GUI (ONT, version 19.05.0) was used for setting appropriate sequencing parameters (analysis protocol, flow cell type, run duration, basecalling mode) and for following the progression of the sequencing runs in real time (number of reads, read length distribution and read quality, and pore activity status).

### 2.5. Bioinformatic Analysis

Raw FAST5 files produced by MinION were basecalled under high accuracy mode using the ONT basecaller *Guppy* (version 3.1.5; used parameters: guppy_basecaller -input_path PATH -save_path PATH -qscore_filtering -min_qscore 7 -flowcell FLO-MIN106 -kit SQK-LSK109 -cpu_threads_per_caller 4 -num_callers 4). Run sequencing statistics were graphically displayed using *MinIONQC* [16]. Basecalled FASTQ files were further processed using *LORCAN* (LOng Read Consensus ANalysis) with parameters -n 20 -m 10 -M 3000 -P 100 -D 50 [17]. The *LORCAN* pipeline was developed for the analysis of barcoded amplicons sequenced with ONT-based methods and was adapted for VP1 based genotyping of enteroviruses in this study. Briefly, the pipeline consists of the following main steps: The basecalled reads are demultiplexed and adapter trimmed off using *porechop* (v0.2.4; [18]). Reads are filtered by length, keeping only those with a length of ±50 bp around the modal sequence length and a maximum of 3000 reads per barcode. Reads are mapped to a custom enterovirus *VP1* reference database (see following section) using *minimap2* [19]. Mapped reads are subsequently binned by genotype level and mapped again to the reference with the highest number of mapping reads within the genotype level. Finally, a consensus sequence is derived using a 50% majority rule consensus. Consensus sequences are used to recruit closely matching sequences in the reference database using *BLASTN*, and a final phylogenetic tree is automatically created with the closest reference sequences using *IQ-TREE* [20]. To enable the analysis of enterovirus *VP1* sequences, a custom *VP1* enterovirus reference database was created by downloading sequences from GenBank using the following criteria ("Enterovirus"(Organism) AND VP1 (All Fields) AND ("380"(SLEN):"10,000"(SLEN)), in May 2019). Sequences were further trimmed to the region of interest (i.e., amplicon sequence without primer binding sites), by using *mothur* [21], following the steps outlined at Create_custom_db/custom_16S.md in [17]. 

The consensus sequences obtained with the *LORCAN* pipeline (based on parameters of up to 3000 reads close to the expected size to be retained per sample; and a minimum number of 100 reads aligned to a reference for the latter to be further considered for sequence consensus generation) were further subjected to consensus sequence improvement using *nanopolish* (version 0.11.1) [22], to determine whether the latter was necessary for enterovirus sequence identification. Multiple sequence alignment was done using *MAFFT* v7.313 [23]. Phylogenetic tree reconstruction and bootstrapping support calculation were done in *MEGA X* [24]. Genotype confirmation was obtained by submitting consensus sequences to the enterovirus genotyping tool *RIVM*. Statistical analysis (regression, correlation, ANOVA) and data visualization was done with the *R* statistical computing environment (version 3.5.0 or later). Sequence identities were calculated using legacy BLAST 2.2.9. 

### 2.6. Data Availability

All sequence data were deposited to the European Nucleotide Archive, under the project reference PRJEB34094.

## 3. Results

We assessed the suitability of the ONT flow cell, “Flongle”, for enterovirus genotyping based on multiplexed amplicons produced from samples of clinical origin. Samples were obtained randomly from enterovirus positive samples referred to IFIK from May 2016 to July 2018. We worked directly from patient material for all samples but two, for which the virus was first propagated in cell culture (Table S1). In order to obtain homogeneous starting material for each sample, PCR amplicons were produced once for each sample. We then associated those amplicons to different barcodes, as follows: The first Flongle sequencing run included 26 barcoded samples. The second Flongle run included the same 26 samples, but this time in duplicates, i.e., using two different barcodes per sample. Therefore, each amplicon sample was sequenced three times and the variability within and between sequencing runs assessed. The first sequencing run contained four additional barcoded samples which were not part of this study and were excluded from further analysis. All amplicons were also sequenced with the Sanger method for comparative purposes. Based on Sanger sequencing, the samples set encompassed 10 different genotypes belonging to enterovirus species A and B (Table S1). 

### 3.1. Flongle Sequencing Run Statistics

**Figure 2:**
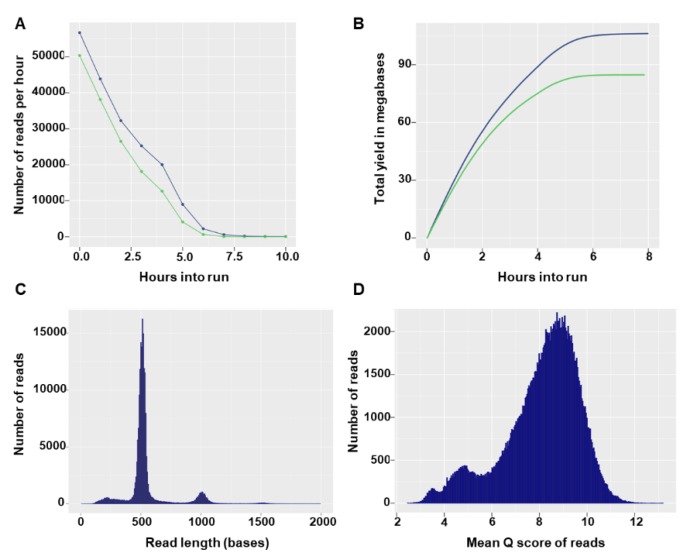
Sequencing run statistics for the first Flongle run. **A**) Number of reads per hour through the progression of the run. Green and blue colors indicate statistics for reads with Q-score ≥ 7 and all reads, respectively. **B**) Total yield in megabases over time throughout the run. **C**) Number of raw reads per read length (bases). **D**) Number of reads per mean Q-score.

Flongle flow cells can have a maximum of 126 available nanopores, with a minimum of 60 pores guaranteed by the manufacturer. In the two sequencing runs, the Flongles had 55 and 60 available pores and after loading of the prepared library the sequencing run started with 27 and 42 active pores, respectively. In both runs, the number of reads per hour decreased rapidly (Figure 2A) and no more notable output was produced after 7 h of sequencing (Figure 2B). The total number of reads generated in the sequencing runs was 190.29 K (135.55 Mbases) and 267.38 K (172.4 Mbases). Read size distributions showed specific modal peak that fit the expected barcoded input amplicon size (Figure 2C). The majority of reads had good quality scores, with 162,217 (85%) and 205,829 (77%) reads having a quality score equal to or higher than 7 in the first and second run, respectively (Figure 2D). Overall, high similarity in yield and run statistics were observed between the two runs (data not shown) and only the first run was used for illustrative purposes.

### 3.2. Sequencing Accuracy Compared to Sanger

FASTQ files containing sequences with quality scores ≥ 7 were processed using the *LORCAN* pipeline. The resulting consensus sequences were polished using the *nanopolish* software. Sequence identities of the raw consensus sequences produced by *LORCAN* and of their polished counterparts were established in comparison to Sanger sequences produced from the same starting amplicons. The average sequence identity of all samples of the first run was 99.85% (standard deviation SD = 0.41). In the second run, the first barcode set had an average of 99.75% identity (SD = 0.46), and the second set an average of 99.80% (SD = 0.23) identity to their Sanger counterparts. The most common differences between the nanopore consensuses and Sanger sequences were a gap or unassigned base (N) in a short homopolymer sequence (4-mer) and these errors were generally observed in different samples associated with the same genotype (data not shown). 

**Figure 3:**
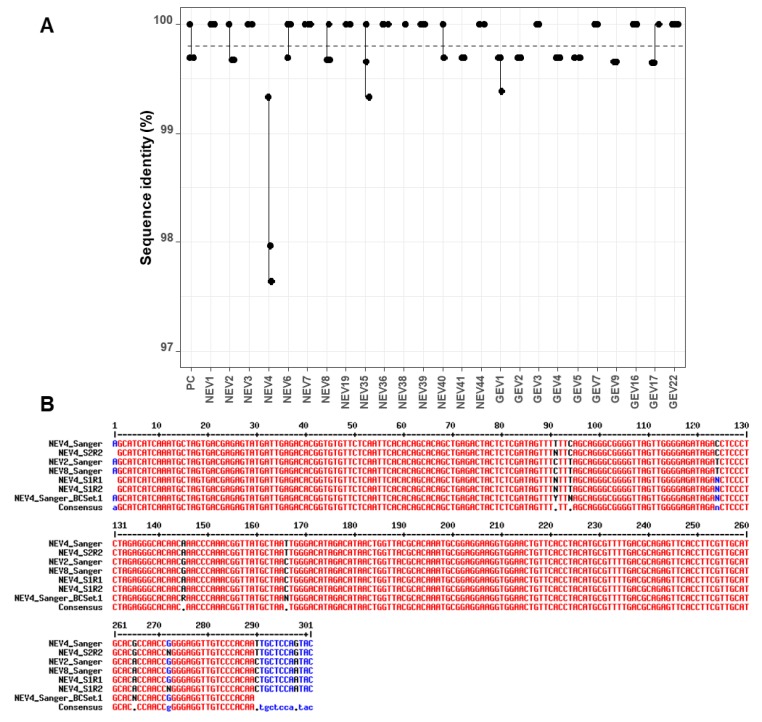
Variability in sequence identity across runs and barcode sets. (**A**) Variability in sequence identity per sample is shown for the consensus sequences of all replicates sequenced during the two Flongle runs. A small amount of horizontal noise was added to the x coordinates to visualize all points. The sequence identity was calculated in reference to the corresponding Sanger sequences. The dashed line indicates the mean sequence identity at 99.80% (SD = 0.37%). (**B**) Alignment of the outlier NEV4 consensus sequences and corresponding Sanger sequences. Additionally, NEV2 and NEV8, which matched the same genotype, were added to the sequence alignment, which was done using *MultAlin* [25]. Sample IDs are abbreviated as follows: S1 = Barcode Set 1, S2 = Barcode Set 2, R1 = Sequencing run 1, R2 = Sequencing run 2. BCSet1 refers to the Sanger sequence of the barcoded amplicon, as opposed to Sanger sequence obtained before barcoding.

The analysis of sequence identity between the three replicates of each sample indicated little variability, with values >99% for all replicates across sequencing runs and barcode sets (Figure 3A). There was, however, one clear outlier (NEV4) within the first set of barcodes, which had several mismatches and unassigned bases compared to the corresponding Sanger sequence (Figure 3B). Additionally, the *LORCAN* consensuses of NEV4 in set 1 in both runs were also the only cases where *nanopolish* correction appeared to introduce more errors rather than improving the sequences (drop of sequence identity from 98.0% to 97.3% and 97.6% to 97.0% for the two runs; Table S2) by introducing additional gaps and a mismatch. These results were consistent between runs of the first set of barcodes, but not observed in the second set of barcodes. 

To investigate this discrepancy in nanopore consensuses, Sanger sequencing of the NEV4 barcoded amplicon after PCR barcoding in the ONT protocol was also performed. Unassigned bases were also evidenced at the same positions (Figure 3B), suggesting the variability might originate from the barcoding PCR step rather than the sequencing run. Another explanation for the discrepancy may be a contamination with NEV2 or NEV8 samples during the barcoding PCR step, because those latter sequences displayed the same sequence variability as in NEV4 nanopore consensuses (comparison of NEV2_Sanger and NEV8_Sanger, and NEV4_S1R1 and NEV4_S1R2 at positions 94 and 166 in Figure 3B). If this scenario is true, lower sequence identity in Figure 3A for NEV4 nanopore consensuses would be explained by the fact that the NEV4 nanopore consensuses were compared to a wrong Sanger reference sequence due to a probable cross-contamination during the barcoding PCR step of Set 1. A closer look at the data indicated that, even though the *LORCAN* generated consensuses from NEV4 displayed an average sequence identity to NEV2 (or NEV8) Sanger sequences of 98.84% for both sequencing runs and only of 97.805% to NEV4 Sanger sequence, the NEV4 nanopore consensuses did not perfectly match any of the NEV2 or NEV8 Sanger reference sequences (Figure 3B). Therefore, the cross-contamination hypothesis for NEV4 is likely, but not completely established at this stage. 

Overall, there was a significant difference in sequence identity when the factors “Run number”, “barcode set”, and “genotype” were evaluated (multivariable linear regression, ANOVA, *F* = 3.236, *P* = 0.001). Significant differences were uniquely due to the factor “genotype” (*F* = 3.7577, *P* = 0.0005), while the factors “barcode set” (*F* = 0.007, *P* = 0.978) and “Run number” (*F* = 1.2579, *P* = 0.2661) were not significant. Although *nanopolish* was able to improve the consensus sequences in a few cases, overall it was not able to make a significant improvement in sequence identity for any of the replicated samples (two-sided t-test, *P > 0.05* for each set). There was also no significant relationship between the number of reads that were used to build the consensus and the sequence identity (ANOVA, *F* = 1.5188, *P* = 0.2216; Pearson correlation coefficient 0.1399).

### 3.3. Phylogenetic Relatedness of Consensus Sequences 

**Figure 4:**
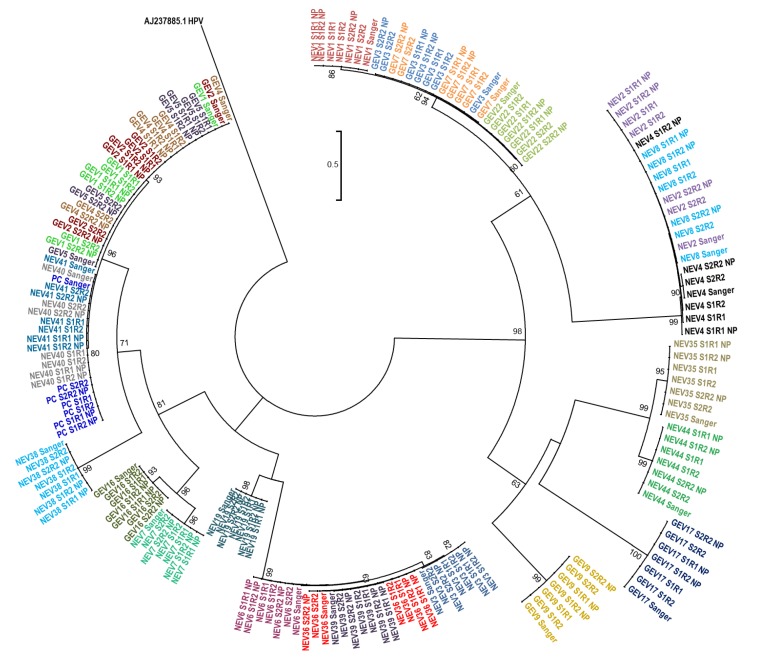
Phylogenetic analysis of all consensus sequences. Nanopore consensus sequences from the two Flongle sequencing runs produced by *LORCAN* with or without *nanopolish* correction, and their corresponding Sanger sequences were aligned with *MAFFT* and subjected to phylogenetic reconstruction. The evolutionary relationships were inferred using the Neighbor-Joining method and the optimal tree with the sum of branch length = 9.910506 is displayed. The percentage of replicate trees in which the associated taxa clustered together in the bootstrap test (1000 replicates) are shown next to the branches. The tree is drawn to scale, with branch lengths in the same units as those of the evolutionary distances used to infer the phylogenetic tree. The evolutionary distances were computed using the maximum composite likelihood method and are in the units of the number of base substitutions per site. The analysis involved 183 nucleotide sequences. All codon positions were included in the analysis, and positions containing gaps and missing data were eliminated, leaving a total of 252 positions (mostly determined by the length of the shorter sequences; Table S3) in the final alignment. Sample ID are abbreviated as follows: S1 = Barcode Set 1, S2 = Barcode Set 2, R1 = Sequencing run 1, R2 = Sequencing run 2, NP = *nanopolish*. Every sample is colored by sample identity to visualize variability of clustering. The tree was rooted based on the *VP1* sequence of human poliovirus 1 (AJ237885.1).

We further evaluated whether the sequences generated by nanopore sequencing using Flongle or by Sanger sequencing produced evolutionary related clusters. A phylogenetic tree including all three Flongle sequencing replicates and the Sanger sequences was constructed (Figure 4). All sequences clustered together with their corresponding Sanger sequence and the results were consistent across all three replicated sample sets (Figure 4).

### 3.4. Sequencing Costs, Turnover Time

We estimated the sequencing cost on Flongle at around $10 per sample when multiplexing 26 amplicon samples. Those costs include costs for the flow cell, quality control (DNA concentration and fragment sizing), and library preparation with plasticware, reagents, and barcoding tailed primers. We demonstrated that sequence quality and taxonomic positioning were not affected by doubling the number of samples (i.e., up to 52) multiplexed together per Flongle sequencing run. Therefore, our estimated sequencing cost may be in the range of $7 per sample, or even lower if more samples are combined together. 

Alternatively, if the use of tailed primers and barcoding PCR is not possible, the native barcoding kits from ONT with a ligation based barcoding approach can be used. Then, cost estimation would be around $12 per sample, with the limitation that only up to 24 samples maximum may be multiplexed together in the same library, which is enabled by using the two native barcoding expansion kits (ONT EXP-NBD104, EXP-NBD114). The turnover time from patient samples to final output (including RNA extraction, reverse transcription and PCR, library preparation, sequencing time, automatic sequence analyses, and reporting) may be estimated at around 22.5 h for each multiplexed Flongle run (Figure 1).

## 4. Discussion

In this proof-of-concept study, we describe the successful application of the new Flongle flow cell for enterovirus genotyping via nanopore sequencing. We were able to correctly identify enterovirus genotypes in all 26 randomly selected clinical samples known to be enterovirus positive using multiplexed amplicon sequencing on Flongles. We observed a high sequencing accuracy when comparing the sequence identity of the resulting consensus sequences with Sanger sequencing, which ranged from 97% to 100%, and the average of the 26 samples was >99.7% identical when considering all replicates. The phylogenetic analysis of the resulting consensus sequences indicated that the Flongle nanopore sequencing provides sufficiently accurate results for correct clustering of sequences, as all sequences grouped together with their Sanger counterparts. These results were also achieved when combining the set twice using different barcodes and therefore combining 52 samples on one flow cell. 

It has been shown previously that nanopore sequencing using regular flow cells can be a useful method for sequencing short amplicons [10,26], and many of the same advantages also apply to Flongles, namely the low capital cost investment for the sequencing device and its portability, making the approach well-suited for diagnostic applications. For instance, nanopore sequencing has been applied for the genotyping of Newcastle disease virus, where >98% identity to Illumina MiSeq was achieved within 7 mins of sequencing and was able to resolve mixed infections of two genotypes from clinical samples [10]. As the regular flow cells have many nanopores available and are designed for larger scale experiment, sufficient sequencing depth of amplicons can be achieved very quickly and due to the real-time nature of nanopore sequencing the run can be stopped after a short time if the desired sequencing depth has been obtained. The flow cell can be reused with a different set of barcodes for another run. The Flongle, in contrast, currently about five times cheaper than a regular flow cell, is designed for single use and has fewer nanopores available, which may therefore increase the sequencing run time substantially. Although a Flongle may be suited for smaller sequencing projects, we demonstrated here that multiplexing on a Flongle may further present significant cost reduction for simple assays, such as amplicon sequencing, without deterioration of the sequencing quality. The turnover time from patient samples to final report was estimated at around 22.5 h for each batch of samples. Since we privileged raw data acquisition over fast turnaround time in this study, we performed basecalling offline using the high accuracy option of the basecaller. Shorter turnaround time could still be obtained by performing live basecalling of the sequencing signal generated by the Flongle.

Using the PCR barcoding kit, the number of samples can in theory be scaled up to 96 per flow cell. The disadvantage, however, can be that it requires another (although short) PCR step for barcoding, thus increasing the chances of PCR cross-contamination and adding to the duration of the overall procedure, which would be better addressed by using automation instead of manual library preparation. Additionally, tailed primers need to be used to generate the amplicons, which may require the PCR conditions to be optimized. In our hands, tailed primers often demonstrate lower sensitivity as compared to the original, unmodified primers (data not shown). On the other hand, the Flongle protocol that we used required very little input, and weak positive samples could also be included. Another source of error may originate from cross-contamination between barcodes, with an estimated amount of up to 0.056% of total reads with incorrect barcodes in multiplex sequencing data [27]. In our approach, we limited the analysis to the 3000 reads whose lengths were closest to the length distribution mode and further required that a least 100 reads mapped to a reference sequence to further derive a consensus sequence from the reads. We also required that *porechop* be used with the more stringent option "require_two_barcodes" for demultiplexing. Those conditions overall may help address the issue with low amount of cross-barcode contamination that may be present. 

One of the main limitations of nanopore sequencing is its still relatively high error rate per base in raw reads as compared to second generation sequencing techniques or to Sanger sequencing. Despite sequence consensus generation using *LORCAN*, which should remove random errors provided enough reads were used to generate the consensus (i.e., at least 100 reads per consensus; data not shown), we did not always achieve 100% identity to the corresponding Sanger sequence, and the main source of error was found to be located in short homopolymer regions: a systematic error known for sequencing using R9.4.1 nanopores [28]. *Nanopolish* is sometimes able to correct this kind of error, although for this dataset it did not make a significant difference in terms of sequence accuracy or phylogenetic positioning. Therefore, the consensus sequences produced by the *LORCAN* pipeline alone were found to be already of sufficient high quality for genotyping applications, even when multiplexing several amplicon samples on the same Flongle flow cell. We did not perform real-time processing of the sequencing data being accumulated on the sequencing computer, and as such, faster turnover time may easily be attained in the future by using simultaneous data acquisition and bioinformatic treatment of the information (e.g., [29]).

With the introduction of the Flongle, Oxford Nanopore Technologies provides a new flow cell that allows scaling down experiments, and which may be ideally suited for fast and cheap amplicon sequencing. Flongle nanopores offer sufficient sequencing accuracy to pool together multiple samples while allowing the possibility of resolving mixed amplicons via the further bioinformatic analyses of each single sequenced molecule, which is not feasible via Sanger sequencing. Consensus sequences obtained via nanopore sequencing may also be longer than their Sanger counterparts, as the latter typically lack tens of bases at the 5’ and 3’ end due to lower base quality, especially when sequencing long amplicons. Further experiments to evaluate the sensitivity of the approach are still required. 

## 5. Conclusions

Overall, we were able to show that we can achieve sequencing accuracy high enough for enterovirus species identification, phylogenetic analyses and genotyping, suggesting a promising future for applications based on amplicon sequencing and for the detection and surveillance of enteroviruses and other pathogens in routine diagnostic laboratories.

## Supplementary Materials

The following are available online at https://www.mdpi.com/2073-4425/10/9/659/s1, Table S1: Sample description and number of reads per barcode for each sequencing run, Table S2: Evaluation of sequencing accuracy, Table S3: Length and BLAST matches of consensus sequences produced by *LORCAN*, Text S1: Library preparation protocol.
