# Supplementary material for: Rapid and Cost-Efficient Enterovirus Genotyping from Clinical Samples Using Flongle Flow Cells
Source: Genes (Basel). 2019 Aug 29;10(9):659. doi: 10.3390/genes10090659 (PMC6770998; doi:10.3390/genes10090659)
Supplement: Supplementary file 1 [file genes-10-00659-s001.zip › 2_Gradel_SI_files (resubmited after reviewers)/SupplementaryText S1.pdf]

Barcoding PCR Protocol: from **1D PCR barcoding (96) amplicons (SQK-LSK109),** PBAC96_9069_v109_revI_23May2018 – Oxford Nanopore Technologies

- In a 0.2 ml 96 well plate, set up a barcoding PCR reaction as follows for each library:
  - 1 µl PCR Barcode (one of BC1-BC96, at 10 µM)
  - 24 µl 0.5 nM first-round PCR product
  - 25 µl LongAmp Taq 2x master mix
- Mix by pipetting.
- Seal the plate with adhesive film or PCR strip caps and briefly spin down in a plate spinner.
- Amplify using the following cycling conditions:
  - Initial denaturation 3 mins @ 95 °C (1 cycle)
  - Denaturation 15 secs @ 95 °C (15 (b) cycles)
  - Annealing 15 secs (a) @ 62 °C (a) (15 (b) cycles)
- Extension dependent on length of target fragment (d) @ 65 °C (c) (12-15 (b) cycles)
- Final extension dependent on length of target fragment (d) @ 65 °C (1 cycle)
- Hold @ 4 °C
- Purify the barcoded DNA using standard methods which are suitable for the fragment size.
- Quantify the barcoded library using standard techniques, and pool all barcoded libraries in the desired
- ratios in a 1.5 ml DNA LoBind Eppendorf tube.
- Prepare 1 µg of pooled barcoded libraries in 47 µl Nuclease-free water.
- This pooled library is now ready to be end-repaired and adapted for nanopore sequencing.

Oxford Nanopore Technologies

Genomic DNA by Ligation (SQK-LSK109) – Flongle

Version: GDF_9078_v109_revN_16Oct2018

DNA repair and end-prep

- Thaw DNA CS (DCS) at RT, spin down, mix by pipetting, and place on ice.
- Prepare the NEBNext FFPE DNA Repair Mix and NEBNext End repair / dA-tailing Module reagents in accordance with manufacturer's instructions, and place on ice.
- In a 0.2 ml thin-walled PCR tube, mix the following:
  - 0.5 µl DNA CS
  - 23.5 µl DNA
  - 1.75 µl NEBNext FFPE DNA Repair Buffer
  - 1 µl NEBNext FFPE DNA Repair Mix
  - 1.75 µl Ultra II End-prep reaction buffer
  - 1.5 µl Ultra II End-prep enzyme mix
- Mix gently by flicking the tube, and spin down.
- Using a thermal cycler, incubate at 20° C for 5 minutes and 65° C for 5 mins.

AMPure XP bead clean-up:

- Prepare the AMPure XP beads for use; resuspend by vortexing.
- Transfer the DNA sample to a clean 1.5 ml Eppendorf DNA LoBind tube.
- Add 30 µl of resuspended AMPure XP beads to the end-prep reaction and mix by flicking the tube.
- Incubate on a Hula mixer (rotator mixer) for 5 minutes at RT.
- Prepare 500 µl of fresh 70% ethanol in Nuclease-free water.
- Spin down the sample and pellet on a magnet. Keep the tube on the magnet, and pipette off the supernatant.
- Keep on magnet, wash beads with 200 µl of freshly prepared 70% ethanol without disturbing the pellet.
- Remove the 70% ethanol using a pipette and discard.
- Repeat the previous step.
- Spin down and place the tube back on the magnet. Pipette off any residual ethanol. Allow to dry for ~30 seconds, but do not dry the pellet to the point of cracking.
- Remove the tube from the magnetic rack and resuspend the pellet in 31 µl Nuclease-free water. Incubate for 2 minutes at RT.
- Pellet the beads on a magnet until the eluate is clear and colourless.
- Remove and retain 31 µl of eluate into a clean 1.5 ml Eppendorf DNA LoBind tube.
- Quantify 1 µl of eluted sample using a Qubit fluorometer.
- Take forward the repaired and end-prepped DNA into the adapter ligation step. However, at this point it is also possible to store the sample at 4° C overnight.

Adapter ligation and clean-up

- Although the recommended 3rd party ligase is supplied with its own buffer, the ligation efficiency of Adapter Mix (AMX) is higher when using Ligation Buffer supplied within SQK-LSK109.
- Spin down Adapter Mix (AMX) and T4 Ligase, and place on ice.
- Thaw Ligation Buffer (LNB) at RT, spin down and mix by pipetting. Due to viscosity, vortexing this buffer is ineffective. Place on ice immediately after thawing and mixing.
- Thaw the Elution Buffer (EB) at RT, mix by vortexing, spin down and place on ice.
- To enrich for DNA fragments of 3 kb or longer, thaw one tube of Long Fragment Buffer (LFB) at RT, mix by vortexing, spin down and place on ice.
- To retain DNA fragments of all sizes, thaw one tube of Short Fragment Buffer (SFB) at RT, mix by vortexing, spin down and place on ice.
- In a 1.5 ml Eppendorf DNA LoBind tube, mix in the following order:
  - 30 µl DNA sample from the previous step
  - 12.5 µl Ligation Buffer (LNB)
  - 5 µl NEBNext Quick T4 DNA Ligase
  - 2.5 µl Adapter Mix (AMX)
- Mix gently by flicking the tube, and spin down.
- Incubate the reaction for 10 minutes at RT.

AMPure XP bead clean-up:

- Prepare the AMPure XP beads for use; resuspend by vortexing.
- Add 20 µl of resuspended AMPure XP beads to the reaction and mix by flicking the tube.

- Incubate on a Hula mixer (rotator mixer) for 5 minutes at RT.
- Spin down the sample and pellet on a magnet. Keep the tube on the magnet, and pipette off the
- supernatant.
- Wash the beads by adding either 125 µl Long Fragment Buffer (LFB) or 125 µl S Fragment Buffer (SFB). Flick the beads to resuspend, then return the tube to the magnetic rack and allow the beads to pellet. Remove the supernatant using a pipette and discard.
- Repeat the previous step.
- Spin down and place the tube back on the magnet. Pipette off any residual supernatant. Allow to dry for
- ~30 seconds, but do not dry the pellet to the point of cracking.
- Remove the tube from the magnetic rack and resuspend pellet in 7 µl Elution Buffer (EB). Incubate for 10 minutes at RT. For high molecular weight DNA, incubating at 37° C can improve the recovery of long fragments.
- Pellet the beads on a magnet until the eluate is clear and colourless.
- Remove and retain 7 µl of eluate into a clean 1.5 ml Eppendorf DNA LoBind tube.
- Remove and retain the eluate which contains the DNA library in a clean 1.5 ml Eppendorf DNA LoBind tube
- Dispose of the pelleted beads
- Quantify 1 µl of eluted sample using a Qubit fluorometer.

The prepared library is used for loading into the flow cell. Store the library on ice until ready to load.

We recommend loading 3-20 fmol of this final prepared library onto the flow cell. Loading more than 50 fmol can have a detrimental effect on throughput. Dilute the library in EB if required.

Loading the Flongle flow cell

IMPORTANT: It is absolutely imperative that the metal contact pads at the back of the Flongle flow cell are not contacted with anything other than the corresponding contact pads of the Flongle adapter.

The anatomy of the Flongle flow cell is important to understand in order to flush the flow cell for library preparation loading.

IMPORTANT: The Sequencing Tether (SQT) will NOT be required in the flushing of the Flongle flow cell or in the making of the Sequencing Mix.

IMPORTANT: Thaw the Sequencing Buffer (SQB), Loading Beads (LB), Flush Buffer (FB) and Flush Tether (FLT) at RT before placing the tubes on ice as soon as thawing is complete.

- Mix the Sequencing Buffer (SQB), Flush Buffer (FB) and Flush Tether (FLT) tubes by vortexing, spin down then return to ice.
- Place the Flongle adapter into the MinION or appropriate GridION position that flow cell will be run from.

- Place the flow cell into the flow cell adapter, ensuring the terminal connectors align between the flow cell and adapter.

IMPORTANT: Flongle flow cells require priming with the components of the Flow Cell Priming Kit (EXP-FLP002).

- In a fresh 1.5 ml DNA-LoBind Eppendorf, mix 117 µl of FB with 3 µl of FLT and mix by pipetting.
- Remove the seal tab from the Flongle flow cell, up to a point where the SpotON port is exposed.
- Exposing the SpotON sample port.

IMPORTANT: Set P100 to 100 µl, mix the combined Flush solutions and aspirate 100 µl of this. Add this to the Flongle sample port using the instructions and video called 'Flongle Add Sample' shown in the orange box below.

Vortex the vial of Loading Beads (LB). Please note that the beads settle quickly, so immediately prepare the Sequencing Mix in a fresh 1.5 ml DNA-LoBind Eppendorf for loading the Flongle, as follows:

  - 15 µl Sequencing Buffer (SQB)
  - 10 µl Loading Beads (LB), mixed immediately before use
  - 5 µl DNA library

- Set pipette to 30 µl, mix your sample and aspirate the pipette to take up 30 µl of Sequencing Mix (Step 7); this will contain your DNA library.

IMPORTANT: Check there are no air gaps at the pipette tip and sample port then vertically insert pipette tip into sample port and distribute slowly into the SpotON sample port.

- Seal Flongle flow cell using adhesive on the seal tab.
- Replace the sequencing platform lid on the MinION or GridION
