# Supplementary material for: Rapid and Cost-Efficient Enterovirus Genotyping from Clinical Samples Using Flongle Flow Cells
Source: Genes (Basel). 2019 Aug 29;10(9):659. doi: 10.3390/genes10090659 (PMC6770998; doi:10.3390/genes10090659)
Supplement: Supplementary file 1 [file genes-10-00659-s001.zip › 2_Gradel_SI_files (resubmited after reviewers)/Table S1.pdf]

						Number of reads					
						RUN 1 Set 1		RUN 2 Set1		RUN 2 Set2	
**Sample Name**	**Referral Date**	**Patient material**	**C_t value**	**Barcode Set 1**	**Barcode Set 2**	Total	Closest to the modal length	Total	Closest to the modal length	Total	Closest to the modal length
PC	05.09.2016	Stool/cell culture	25.1	BC1	BC18	3749	3000	1682	1612	2419	2307
NEV1	24.05.2016	Stool	25	BC2	BC19	3967	3000	1910	1561	2939	2492
NEV2	15.06.2016	Stool	22.3	BC3	BC20	2216	2118	1315	1266	2042	1971
NEV3	23.06.2016	Stool	21.6	BC4	BC21	2520	2090	1041	860	1946	1709
NEV4	30.06.2016	Stool	NA	BC5	BC22	2854	2612	1131	1019	2195	2049
NEV6	05.07.2016	Stool	NA	BC7	BC24	3062	2948	1290	1232	1963	1842
NEV7	18.07.2016	Stool	27.8	BC8	BC30	3418	3000	1596	1526	3172	3000
NEV8	19.07.2016	Stool	29.6	BC9	BC31	2640	2431	1232	1161	2227	2112
NEV19	15.07.2018	Stool	30.2	BC12	BC34	2069	2039	994	968	2475	2417
NEV35	28.07.2016	Stool	17	BC16	BC43	3181	2505	1363	1090	2839	2395
NEV36	13.08.2016	Stool	24	BC17	BC44	2379	2339	1166	1142	1858	1821
NEV38	26.08.2016	Stool	31.6	BC27	BC45	3084	2995	1631	1551	2801	2653
NEV39	27.08.2016	Stool	22.2	BC28	BC46	3667	3000	1573	1409	2498	2296
NEV40	29.08.2016	Stool	26.7	BC29	BC47	3394	2968	1471	1282	2042	1802
NEV41	05.09.2016	Stool	25.1	BC37	BC48	1918	1768	1067	993	1940	1789
NEV44	29.10.2018	Nasopharyngeal swab/cell culture	NA	BC40	BC54	3884	3000	2102	2028	3109	3000
GEV1	15.05.2017	Stool	20.3	BC61	BC55	2056	1848	940	853	3055	2726
GEV2	09.06.2017	Stool	25.34	BC73	BC56	3968	3000	1796	1652	2592	2472
GEV3	09.06.2017	Stool	28.04	BC74	BC57	3712	3000	1714	1557	2447	2191
GEV4	12.06.2017	Stool	28	BC75	BC58	2569	2410	1359	1259	2754	2503
GEV5	22.04.2017	Stool	25.7	BC76	BC59	4059	3000	1810	1730	3234	3000
GEV7	10.07.2017	Stool	23	BC77	BC60	3224	3000	1646	1571	1873	1718
GEV9	18.07.2017	Stool	27.8	BC85	BC71	2211	1924	1179	997	2192	1821
GEV16	13.07.2017	Stool	22.13	BC87	BC68	1924	1659	970	828	3232	2738
GEV17	14.06.2017	Stool	21.3	BC88	BC69	2921	2238	1622	1193	4753	3000
GEV22	06.06.2017	Throat swab	25.27	BC89	BC70	2547	2345	1268	1113	3488	3000

SI Table 1: Sample description and number of reads per barcode for each sequencing run. Two samples were grown in cell culture before nucleic acid extraction. The indicated C_t value from the RT-PCR analysis was always determined from the original material. The barcode numbers refer to the assignment according to PCR Barcoding Expansion 1-96 (EXP-PBC096) kit from Oxford Nanopore Technologies.
