# Supplementary material for: Rapid and Cost-Efficient Enterovirus Genotyping from Clinical Samples Using Flongle Flow Cells
Source: Genes (Basel). 2019 Aug 29;10(9):659. doi: 10.3390/genes10090659 (PMC6770998; doi:10.3390/genes10090659)
Supplement: Supplementary file 1 [file genes-10-00659-s001.zip › 2_Gradel_SI_files (resubmited after reviewers)/Table S2.pdf]

		RUN 1								RUN 2															
		Barcode Set 1								Barcode Set 1								Barcode Set 2							
				LORCAN Consensus			Nanopolish Consensus					LORCAN Consensus			Nanopolish Consensus					LORCAN Consensus			Nanopolish Consensus		
Sample Name	Genotype	Alignment length	Number of reads used for consensus	Seq Ident. (%)	Gaps	N's	Seq Ident. (%)	Gaps	N's	Alignment length	Number of reads used for consensus	Seq Ident. (%)	Gaps	N's	Seq Ident. (%)	Gaps	N's	Alignment length	Number of reads used for consensus	Seq Ident. (%)	Gaps	N's	Seq Ident. (%)	Gaps	N's
PC	Echovirus 30	324	2968	100	0	0	100	0	0	324	1570	99.69	1	0	99.69	1	0	324	2267	99.69	1	0	99.69	1	0
NEV1	Coxsackievirus A16	299	2991	100	0	0	100	0	0	299	1553	100	0	0	100	0	0	299	2484	100	0	0	100	0	0
NEV2	Enterovirus A71	300	2096	100	0	0	100	0	0	300	1250	99.67	0	1	99.67	0	1	300	1935	99.67	0	1	99.67	0	1
NEV3	Echovirus 18	321	2063	100	0	0	100	0	0	321	834	100	0	0	100	0	0	321	1665	100	0	0	100	0	0
NEV4	Enterovirus A71	296 (LC) /299 (NP)	2572	97.97	0	2 N, 4 M	97.32	3	5 M	296 (LC) /298 (NP)	996	97.64	0	3 N, 4 M	96.98	2	6 M, 1 N	300	1999	99.33	0	1	99.67	0	1
NEV6	Echovirus 18	324	2923	100	0	0	100	0	0	324	1206	99.69	0	1	100	0	0	324	1811	100	0	0	100	0	0
NEV7	Echovirus 25	321	2984	100	0	0	100	0	0	321	1512	100	0	0	100	0	0	321	2968	100	0	0	100	0	0
NEV8	Enterovirus A71	300	2413	100	0	0	100	0	0	300	1141	99.67	0	1	99.67	0	1	300	2077	99.67	0	1	99.67	0	1
NEV19	Echovirus 5	324	1912	100	0	0	100	0	0	324	864	100	0	0	100	0	0	324	2159	100	0	0	100	0	0
NEV35	Coxsackievirus A4	297	2426	100	0	0	100	0	0	297	997	99.66	0	1	100	0	0	297	2217	99.33	0	2	99.66	0	1
NEV36	Echovirus 18	321	2315	100	0	0	100	0	0	321	1116	100	0	0	100	0	0	321	1769	100	0	0	100	0	0
NEV38	Coxsackievirus B3	283	2870	100	0	0	100	0	0	283	1431	100	0	0	100	0	0	283	2423	100	0	0	100	0	0
NEV39	Echovirus 18	321	2970	100	0	0	100	0	0	321	1376	100	0	0	100	0	0	321	2217	100	0	0	100	0	0
NEV40	Echovirus 30	324	2934	100	0	0	100	0	0	324	1243	99.69	1	0	99.69	1	0	324	1762	99.69	1	0	99.69	1	0
NEV41	Echovirus 30	324	1739	99.69	0	1	99.69	0	1	324	969	99.69	1	0	99.69	1	0	324	1749	99.69	1	0	99.69	1	0
NEV44	Coxsackievirus A4	297	2930	100	0	0	100	0	0	297	1932	100	0	0	100	0	0	297	2857	100	0	0	100	0	0
GEV1	Echovirus 30	324	1841	99.69	0	1	99.69	0	1	324	850	99.69	1	0	99.69	1	0	324	2709	99.38	1	1	99.38	1	1
GEV2	Echovirus 30	320	2981	99.69	0	1	99.69	0	1	320	1639	99.69	0	1	99.69	0	1	320	2433	99.69	1	0	99.69	1	0
GEV3	Coxsackievirus A16	300	2989	100	0	0	100	0	0	300	1548	100	0	0	100	0	0	300	2175	100	0	0	100	0	0
GEV4	Echovirus 30	324	2397	99.69	1	0	99.69	1	0	324	1242	99.69	1	0	99.69	1	0	324	2476	99.69	1	0	99.69	1	0
GEV5	Echovirus 30	324	2994	99.69	0	1	99.69	0	1	324	1712	99.69	1	0	99.69	1	0	324	2975	99.69	1	0	99.69	1	0
GEV7	Coxsackievirus A16	300	2992	100	0	0	100	0	0	300	1562	100	0	0	100	0	0	300	1704	100	0	0	100	0	0
GEV9	Coxsackievirus A2	297	1809	99.66	0	1	99.66	0	1	297	899	99.66	0	1	99.66	0	1	297	1654	99.66	0	1	99.66	0	1
GEV16	Echovirus E25	321	1621	100	0	0	100	0	0	321	793	100	0	0	100	0	0	321	2578	100	0	0	100	0	0
GEV17	Coxsackievirus A6	288	2212	100	0	0	100	0	0	288	1164	99.65	0	1	100	0	0	288	2918	99.65	0	1	100	0	0
GEV22	Coxsackievirus A16	297	2334	100	0	0	100	0	0	297	1103	100	0	0	100	0	0	297	2986	100	0	0	100	0	0

Table S2: **Sequence comparison of nanopore consensus sequences relative to the Sanger sequence**. For each sample, the genotype assigned to both Sanger and Nanopore consensus sequences is indicated. For each run and barcode set, the sequence identity was calculated from the LORCAN consensus and after applying nanopolish. Additionally, alignment length, the number of gaps and N's (unassigned bases) in the sequence alignments are indicated. M= Mismatch, N= unassigned base, LC = LORCAN, NP= Nanopolish. Sequence Identity (%) is always relative to the Sanger sequence obtained from the same amplicon.
