# Supplementary material for: Rapid and Cost-Efficient Enterovirus Genotyping from Clinical Samples Using Flongle Flow Cells
Source: Genes (Basel). 2019 Aug 29;10(9):659. doi: 10.3390/genes10090659 (PMC6770998; doi:10.3390/genes10090659)
Supplement: Supplementary file 1 [file genes-10-00659-s001.zip › 2_Gradel_SI_files (resubmited after reviewers)/Table S3.pdf]

Sample Name	RUN1			RUN2					
	Barcode Set 1			Barcode Set 1			Barcode Set 2		
	Consensus length	Sequence match to closest reference sequence	Accession number of closest reference sequence	Consensus length	Sequence match to closest reference sequence	Accession number of closest reference sequence	Consensus length	Sequence match to closest reference sequence	Accession number of closest reference sequence
PC	324	309/324 (95.37%)	HG793732.1	323	308/324 (95.06%)	HG793732.1	323	308/324 (95.06%)	HG793732.1
NEV1	300	299/300 (99.67%)	LT577809.1	300	299/300 (99.67%)	LT577809.1	300	299/300 (99.67%)	LT577809.1
NEV2	300	300/300 (100.00%)	MH472702.1	300	299/300 (99.67%)	MH472702.1	300	299/300 (99.67%)	MH472702.1
NEV3	324	313/324 (96.61%)	HG793726.1	323	312/323 (96.59%)	HG793726.1	324	313/324 (96.61%)	HG793726.1
NEV4	300	298/300 (99.33%)	MH084327.1	300	297/300 (99.00%)	MH084327.1	300	293/300 (97.67%)	MH084327.1
NEV6	324	313/324 (96.61%)	LN876160.1	324	312/324 (96.30%)	LN876160.1	324	313/324 (96.61%)	LN876160.1
NEV7	321	315/321 (98.13%)	HG793729.1	321	315/321 (98.13%)	HG793729.1	321	315/321 (98.13%)	HG793729.1
NEV8	300	300/300 (100.00%)	MH472702.1	300	299/300 (99.67%)	MH472702.1	300	299/300 (99.67%)	MH472702.1
NEV19	324	311/324 (95.99%)	HG793679.1	324	311/324 (95.99%)	HG793679.1	324	311/324 (95.99%)	HG793679.1
NEV35	297	/283/297 (95.29%)	JN203507.1	297	283/297 (95.29%)	JN203507.1	297	283/297 (95.29%)	JN203507.1
NEV36	324	/314/324 (96.91%)	LN876160.1	324	314/324 (96.91%)	LN876160.1	324	314/324 (96.91%)	LN876160.1
NEV38	312	299/312 (95.83%)	MF678314.1	312	299/312 (95.83%)	MF678314.1	312	299/312 (95.83%)	MF678314.1
NEV39	324	313/324 96.61%)	LN876160.1	324	313/324 (96.61%)	LN876160.1	324	313/324 (96.61%)	LN876160.1
NEV40	324	309/324 (95.37%)	HG793732.1	323	308/324 (95.06%)	HG793732.1	323	308/324 (95.06%)	HG793732.1
NEV41	324	308/324 (95.06%)	HG793732.1	323	308/324 (95.06%)	HG793732.1	323	308/324 (95.06%)	HG793732.1
NEV44	297	291/297 (97.98%)	KR185978.1	297	291/297 (97.98%)	KR185978.1	297	291/297 (97.98%)	KR185978.1
GEV1	324	314/324 (96.91%)	HG793732.1	323	314/324 (96.91%)	HG793732.1	323	313/324 (96.61%)	HG793732.1
GEV2	324	314/324 (96.91%)	HG793732.1	324	314/324 (96.91%)	HG793732.1	323	314/324 (96.91%)	HG793732.1
GEV3	300	296/300 (98.67%)	MH010203.1	300	296/300 (98.67%)	MH010203.1	300	296/300 (98.67%)	MH010203.1
GEV4	323	314/324 (96.91%)	HG793732.1	323	314/324 (96.91%)	HG793732.1	323	314/324 (96.91%)	HG793732.1
GEV5	324	314/324 (96.91%)	HG793732.1	323	314/324 (96.91%)	HG793732.1	323	314/324 (96.91%)	HG793732.1
GEV7	300	296/300 (98.67%)	MH010203.1	300	296/300 (98.67%)	MH010203.1	300	296/300 (98.67%)	MH010203.1
GEV9	297	287/297 (96.63%)	MH111019.1	297	287/297 (96.63%)	MH111019.1	297	287/297 (96.63%)	MH111019.1
GEV16	321	306/321 (95.33%)	LC201504.1	321	306/321 (95.33%)	LC201504.1	321	306/321 (95.33%)	LC201504.1
GEV17	297	290/297 (97.64%)	MG885750.1	297	289/297 (97.31%)	MG885750.1	297	289/297 (97.31%)	MG885750.1
GEV22	300	297/300 (99.00%)	MH010203.1	300	297/300 (99.00%)	MH010203.1	300	297/300 (99.00%)	MH010203.1

Table S3. Length and BLAST matches of consensus sequences produced by *LORCAN*.
